# One-Minute Synthesis of Size-Controlled Fucoidan-Gold Nanosystems: Antitumoral Activity and Dark Field Imaging

**DOI:** 10.3390/ma13051076

**Published:** 2020-02-28

**Authors:** Ricardo J. B. Pinto, Daniela Bispo, Carla Vilela, Alexandre M. P. Botas, Rute A. S. Ferreira, Ana C. Menezes, Fábio Campos, Helena Oliveira, Maria H. Abreu, Sónia A. O. Santos, Carmen S. R. Freire

**Affiliations:** 1 Department of Chemistry, CICECO—Aveiro Institute of Materials, University of Aveiro, 3810-193 Aveiro, Portugal; d.bispo@ua.pt (D.B.); cvilela@ua.pt (C.V.); santos.sonia@ua.pt (S.A.O.S.); 2 Phantom-G, Department of Physics, CICECO—Aveiro Institute of Materials, University of Aveiro, 3810-193 Aveiro, Portugal; a.botas@ua.pt (A.M.P.B.); rferreira@ua.pt (R.A.S.F.); 3 Department of Biology & CESAM, University of Aveiro, 3810-193 Aveiro, Portugal; catarinamenezes@msn.com (A.C.M.); f.m.c@ua.pt (F.C.); holiveira@ua.pt (H.O.); 4 ALGAplus—Prod. e Comerc. De Algas e Seus Derivados, Lda., 3830-196 Ílhavo, Portugal; helena.abreu@algaplus.pt

**Keywords:** gold nanoparticles, fucoidan, microwave irradiation, antitumoral activity, darkfield imaging

## Abstract

Gold nanoparticles (AuNPs) are one of the most studied nanosystems with great potential for biomedical applications, including cancer therapy. Although some gold-based systems have been described, the use of green and faster methods that allow the control of their properties is of prime importance. Thus, the present study reports a one-minute microwave-assisted synthesis of fucoidan-coated AuNPs with controllable size and high antitumoral activity. The NPs were synthesized using a fucoidan-enriched fraction extracted from *Fucus vesiculosus*, as the reducing and capping agent. The ensuing monodispersed and spherical NPs exhibit tiny diameters between 5.8 and 13.4 nm for concentrations of fucoidan between 0.5 and 0.05% (w/v), respectively, as excellent colloidal stability in distinct solutions and culture media. Furthermore, the NPs present antitumoral activity against three human tumor cell lines (MNT-1, HepG2, and MG-63), and flow cytometry in combination with dark-field imaging confirmed the cellular uptake of NPs by MG-63 cell line.

## 1. Introduction

Cancer, i.e., the abnormal growth and proliferation of cells, is one of the leading causes of mortality and morbidity worldwide. According to the World Health Organization (WHO), cancer was responsible for 9.9 million deaths in 2018 [1], and the number of cases is anticipated to increase by about 70% over the next two decades. Each cancer type involves a specific treatment procedure that embraces one or more modalities, namely surgery (to remove the tumor), radiotherapy, or chemotherapy. Despite the high cure rates observed when cancer is detected early and if appropriated treatment is provided, most of the presently employed therapies, particularly conventional chemotherapy, are associated with severe side effects, including hair loss, nausea and vomiting, pain, anemia, fertility issues, edema, among many others [2], that strongly affect the patient’s quality of life. In recent decades, the design of more effective alternatives that allows a targeting action, with almost no impact on healthy tissues or organs, has received considerable attention [3]. Of these, nanosystems that combine a therapeutic effect and imaging properties, or that promote intertwined diagnosis and therapy, the so-called nanotheranostics, has opened new avenues for cancer-conquering [4,5,6].

Metal nanoparticles (NPs) are a class of nanomaterials with a panoply of biomedical and therapeutic applications, including cancer therapy and imaging of tumors [7,8]. In particular, gold (Au) nanoparticles owing to their unique physical and optical properties have attracted enormous interest in this realm during the past decades [9,10,11,12]. The colloidal stability of NPs in biological environments and their interactions with cells is strongly influenced by their surface properties, and thus distinct coating strategies of NPs, using small molecules, polymers, or lipids, have been described [13]. Moreover, this methodology could also target the reduction of the toxicity of NPs and improvement of biological functionalities, typically associated with the use of active biomolecules [13]. The synthesis of AuNPs using biomacromolecules, including alginate, starch, cellulose, chitosan, gelatin, collagen and fucoidan, among others, as reducing and stabilizing agents is a well-documented strategy to achieve these goals [14] and, at the same time, overcome the environmental effects of the conventional methodologies that commonly involve the use of harmful reducing agents.

Fucoidan is a natural occurring sulfated marine polysaccharide extracted from brown seaweeds that presents various biological properties, including antiangiogenic, antitumoral, and anti-inflammatory properties [15,16], and because of that has been widely investigated for the development of nanomaterials for biomedical applications [17]. However, only a few number of papers reported the combination of fucoidan and AuNPs for cancer treatment. One of the first studies in this topic involved the synthesis of AuNPs (44 nm average size) using sodium borohydride as the reducing agent and a fucoidan-mimetic glycopolymer as the capping agent [18]. The obtained AuNPs displayed excellent colloidal stability and selective cytotoxicity to human colon cancer cell line (HCT116). Afterward, Manivasagan et al. [19] produced biocompatible AuNPs (82 nm average size) by using naturally occurring fucoidan as the reducing and capping agent, avoiding the use of harmful reducing agents. This study also demonstrated the applicability of these NPs as a carrier for doxorubicin (DOX) and photoacoustic imaging of breast cancer tumors. In a follow-up study, this research team explored similar fucoidan-AuNPs for dual-chemo-photothermal treatment of eye tumors [20]. In another study, size-controlled fucoidan-AuNPs (15-80 nm) were produced by varying the concentration of fucoidan during the synthesis step [21]. These NPs showed anticancer effect against human oral squamous cell carcinoma (HSC3), and its surface modification and conjugation with DOX also improved their effect.

These studies clearly demonstrate the prospective of the partnership between AuNPs and fucoidan in cancer treatment. However, to achieve high antitumoral activities, viz., less than 20% cell viability, the conjugation with other chemotherapeutics (DOX), or the use of high dosages (up to 50 μg mL^−1^ of NPs) was typically required. Moreover, some methodologies are somewhat time-consuming and laborious, e.g., up to 2 h for the synthesis of the AuNPs and 24 h for the conjugation with DOX. Additionally, the number of investigated tumor cell lines is limited, and the imaging properties of fucoidan-AuNPs have been only marginally explored. Thus, some essential traits still need to be tackled envisioning their scale-up production and broad application, viz., the establishment of fast and straightforward procedures for the synthesis of fucoidan-Au nanosystems with controllable size and morphology and improved antitumoral activity against different tumor cell lines.

As a developing heating tool, microwave irradiation has been shown to considerably reduce the reaction times and provide a uniform bulk heating that allows the synthesis of various nanomaterials, including AuNPs [22,23], with defined structures and narrow size distributions. However, to the best of our knowledge, this methodology has never been sightseen as a simple, time-saving approach to fabricate fucoidan-AuNPs for application in cancer therapy.

In this line, in the present study, we report for the first time a one-minute microwave-assisted synthesis of fucoidan-coated AuNPs with controllable size and high antitumoral activity. This is a pioneering achievement with respect to previous methodologies to produce Au-fucoidan NPs. The fucoidan-AuNPs were synthesized, using a fucoidan-enriched fraction extracted from *F. vesiculosus*, and characterized in terms of structure, colloidal stability, antitumoral activity against different cell lines (MNT-1, HepG2, and MG-63), and cellular uptake by flow cytometry and dark field imaging of NPs. The antitumoral activity of Au-fucoidan NPs against these cell lines and their imaging properties by dark-field is also reported here for the first time.

## 2. Materials and Methods

### 2.1. Materials

*Fucus vesiculosus* was collected (January 2014) in Mindelo beach (41°18′38′’N, 8°,43′42′’W), Portugal. The biomass was washed with water to remove salts, epiphytes, and/or microorganisms and dried at 25 °C until reaching a total moisture content of 12–14%. Algae samples were transformed into flakes (1–2 mm) with a knife mill (Retsch SM100, Haan, Germany), packed and stored in airtight bags at the ALGAplus warehouse. The milled algae samples were then washed with a solvent mixture (1 g per 20 mL) of chloroform and methanol (2:1 v/v) under stirring for 20 min, centrifuged at 2500 rpm during 20 min, and dried at 40 °C in a vacuum drying oven.

Gold(III) chloride trihydrate (≥99.9% trace metals basis), dimethyl sulfoxide (DMSO), paraformaldehyde, and Triton X-100 were purchased from Sigma-Aldrich (St. Louis, MO, USA). Phosphate buffer saline (PBS, pH 7.4), Dulbecco′s modified Eagle′s medium (DMEM), fetal bovine serum (FBS), L-glutamine, penicillin, streptomycin and amphotericin B were supplied by Gibco^®^ (Life Technologies, Grand Island, NY, USA), 3-(4,5-dimethylthiazol-2-yl)-2,5-diphenyltetrazolium bromide (MTT, 98%) was purchased from Sigma-Aldrich, and 4′,6-diamidino-2-phenylindole (DAPI)-containing Vectashield mounting medium was acquired from Vector Labs. Ultra-purified water (Type 1, 18.2 MΩ·cm at 25 °C) was obtained by a Simplicity^®^ Water Purification System (Merck, Darmstadt, Germany). Human osteosarcoma cell line MG-63 was a kind gift by INEB, University of Porto, Portugal. The HepG2 cell line, a liver hepatocellular carcinoma cell line, was obtained from the European Collection of Authenticated Cell Cultures (ECACC, Salisbury, UK) and supplied by Sigma-Aldrich. MNT-1 cells were kindly provided by Doctor Manuela Gaspar (iMed.ULisboa, Lisbon, Portugal).

### 2.2. Microwave-Assisted Extraction (MAE) of Fucoidan from F. Vesiculosus

The MAE of fucoidan-rich fraction from *F. vesiculosus* followed the methodology described by Rodriguez-Jasso et al. [24]. About 0.4 g of macroalgae sample was suspended in water, with a solid-liquid ratio of 1:25 (w/v). The extraction was performed in a Monowave 300 (Anton Paar, Graz, Austria) equipment, at 172 ℃, for 1 min. The samples were cooled on ice and then centrifuged at 4000 rpm during 5 min). The aqueous extract was mixed with a 1% (w/v) CaCl_2_ aqueous solution, in a solid-liquid ratio of 1:1 (v/v), and maintained overnight at 4 ℃, to precipitate alginate. This was separated by filtration, and ethanol was added to the filtrate (1:2, v/v) and maintained at 4 ℃ for 8 h. The fucoidan-rich fraction (MWF) was obtained after centrifugation (4000 rpm, 5 min) and dried at room temperature.

### 2.3. Microwave-Assisted Synthesis of Fucoidan-AuNPs

The microwave (MW)-assisted synthesis of fucoidan-AuNPs was carried out on a Monowave 300 equipment (Anton Paar, firmware version 2.0). Total of 145 µL of HAuCl_4_.3H_2_O solution (17.2 mM) was added to microwave vials with 5 mL of the three distinct concentrations of the fucoidan-rich fraction (0.5, 0.1, and 0.05% w/v). The mixtures were heated at 120 ℃ for 1 min. After the reaction, the obtained fucoidan-AuNPs were centrifuged for 1 h at 15,000 rpm and 4 ℃, sonicated and washed three times with ultra-purified water, and finally stored at 4 ℃ until usage.

### 2.4. Structural Characterization of the Fucoidan-Enriched Fraction and Fucoidan-AuNPs

The sulfate content of the fucoidan-enriched fraction was determined by elemental analysis. The fucoidan-rich fraction was grounded with a mortar and analyzed (about 2–3 mg) in a Leco TruSpec 630-200-200 CHNS elemental analyzer (LECO Corporation, St. Joseph, MI, USA), in order to assess the carbon (C), hydrogen (H), nitrogen (N), and sulfur (S) contents. The sulfate content of the sample was calculated by the following equation: sulfate content (%) = 3.22 × S (%), where S (%) is the S content, as proposed by several authors [25,26].

Total sugars were determined by the phenol-sulfuric acid method, following the methodology described by DuBois et al. [27] where D(+)-glucose was used as standard. An aqueous solution of the fucoidan-rich fraction (0.05% w/v) was prepared and diluted as necessary. The UV absorbance measurements were performed in a Shimadzu UV-1800 spectrophotometer (Shimadzu Corp., Kyoto, Japan) at λ = 490 nm. Triplicate measurements were carried out.

Optical spectra of the fucoidan-AuNPs were recorded by a Thermo Scientific Evolution 220 spectrophotometer (Thermo Fisher Scientific, Waltham, MA, USA) using 100 scans min^−1^ with a bandwidth of 2 nm and an integration time of 0.3 s.

The Fourier transform infrared (FTIR) spectra of fucoidan-enriched fraction extracted from *F. vesiculosus* and fucoidan-Au colloidal in the form of KBr pellet were collected by a Mattson 7000 spectrometer using 256 scans at a resolution of 4 cm^−1^ and with a signal gain of 1.

Transmission electron microscopy (TEM) images were obtained by a Hitachi H-9000 (Hitachi High-Technologies Corporation, Tokyo, Japan) operating at 300 kV. Scanning transmission electron microscopy (STEM) images were acquired by a field-emission gun (FEG) SEM Hitachi SU70 microscope operated at 15 kV. Samples for microscopy analysis were prepared by placing a washed colloid drop directly onto a carbon-coated copper grid and then allowing the solvent to evaporate. The average diameter of the NPs was determined by measuring over 100 NPs for each STEM image with the Fiji image processing software.

### 2.5. Colloidal Stability of Fucoidan-AuNPs

The colloidal stability of the fucoidan-AuNPs was evaluated in five different mediums, namely ultra-purified water, HCl (0.01 M, pH 2.1), NaOH (0.01 M, pH 12.0), PBS (pH 7.4), and DMEM. Typically, 100 µL of the fucoidan-AuNPs 0.1% (w/v) was added to the vials already with 2.9 mL of the distinct mediums. The colloidal suspensions were placed under mechanical stirring during 48 h at room temperature, and the UV-Vis spectra at specific times (0, 6, 24, and 48 h) were recorded. All assays were performed in triplicate. After 48 h, the NPs in each medium were centrifuged for 15 min at 15,000 rpm (4 ℃), sonicated and washed three times with ultra-purified water and analyzed by STEM as previously described.

### 2.6. Cell Culture

The cell lines were cultured in DMEM supplemented with 10% fetal bovine serum, 2 mM L-glutamine, 100 U mL^−1^ penicillin, 100 µg mL^−1^ streptomycin, and 2.5 µg mL^−1^ amphotericin B. Cells were incubated in a humidified atmosphere of 5% CO_2_ at 37 ℃. Sub confluent cells were trypsinized with trypsin-EDTA (0.25% trypsin, 1 mM EDTA) when monolayers reached 70% confluence.

### 2.7. Cytotoxicity Evaluation

Cell viability was determined by the colorimetric MTT assay, which measures the formation of purple formazan in viable cells [28]. Cells were seeded in 96-well plates and after 24 h, medium was replaced with fresh medium containing fucoidan extract (0, 0.25, 0.5, 1.0, 2.5, and 5 mg mL^−1^) or fucoidan-AuNPs (0, 2.5, 5.0, 10.0, 15.0, and 26.0 µg mL^−1^). Cell viability was measured after 24, 48, and 72 h. After that, 50 µL of MTT reagent (1 mg mL^−1^) in PBS was added to each well and incubated for 4 h at 37 ℃, and 5% CO_2_. The medium was then removed, and 150 µL of DMSO was added to each well for crystals solubilization. The optical density of the reduced MTT was measured at 570 nm in a microtiter plate reader (Synergy HT Multi-Mode, BioTeK instruments, Winooski, VT, USA), and the cell metabolic activity (MA, a usual marker for cell viability) was calculated as MA (%) = ((Abs _sample_−Abs _DMSO_)/(Abs _control_−Abs _DMSO_)) × 100. Three independent assays were performed with at least three technical replicates each and the results compared with the control (incubated with culture medium). From the MTT results, the concentrations of 5 and 12 µg mL^−1^ of fucoidan-AuNPs were selected for the following assays.

### 2.8. Uptake Potential by Flow Cytometry

The uptake potential of fucoidan-AuNPs by MG-63 cells was assessed by flow cytometry (FCM), as previously described by Suzuki et al. [29] and Bastos et al. [30]. Briefly, cells were seeded in 6-well plates, and after fucoidan-AuNPs exposure for 24 h, they were trypsinized, collected to FCM tubes, and analyzed by FCM. Two parameters, namely forward scatter (FS), which gives information on the particle’s size, and side scatter (SS), which provides information on the complexity of particles, were measured in an Attune^®^ Acoustic Focusing Cytometer (Thermo Scientific, Waltham, MA, USA) equipped with a 488 nm laser. For each sample, 5000–20,000 cells were analyzed at a flow rate of about 300 cells s^−1^.

For MTT assay and cellular uptake by flow cytometry, the statistical significance between control and exposed cells was performed by one-way ANOVA, followed by Dunnet and Dunn’s method (as parametric and non-parametric test, respectively), using Sigma Plot 12.5 software (Systat Software Inc.).

### 2.9. Dark Field Imaging

MG-63 cells were grown on glass coverslips and cultured in the presence of 12 µg mL^−1^ fucoidan-AuNPs dispersed in culture medium for 24 h. Cells were fixed with a 4% paraformaldehyde in PBS for 10 min, permeabilized with a 0.1% Triton X-100/PBS solution. Following washes with PBS and deionized water, coverslips were mounted onto the glass slides with DAPI-containing Vectashield mounting medium.

The microscopic images were recorded using an Olympus BX51 microscope (50× objective) (Olympus, Tokyo, Japan) equipped with a digital CCD camera (Retiga 4000R, QImaging) used to capture the microphotographs. The dark field images were acquired under white light illumination by replacing the standard microscope condenser by the CytoViva enhanced dark field illumination system (CytoViva, Auburn, AL, USA). For the images under white light illumination and UV irradiation, a DC regulated illuminator (DC-950, Fiber-Lite) and a LED light (LLS-365, Ocean Optics, emission at 365 ± 25 nm) were used, respectively.

The hyperspectral images were recorded with a hyperspectral imaging system from CytoViva, accoupled to the Olympus BX51 microscope, that includes a digital camera (Pixelfly USB, PCO) coupled to a spectrograph (V10E 2/3′’, Specim, 30 μm slit, nominal spectral range of 400–1000 nm and nominal spectral resolution of 2.73 nm). Each pixel field-of-view on the hyperspectral images corresponds to 258 × 258 nm^2^ on the samples’ plane. The hyperspectral scanning is vertical, and each image results from 696 lines, using an exposure time of 3 s for each line. All the hyperspectral data were acquired and analyzed using ENVI 4.8 software, and the spectra were corrected using the tool Calibration and Correction of the ENVI 4.8 software.

## 3. Results and Discussion

**Figure 1:**
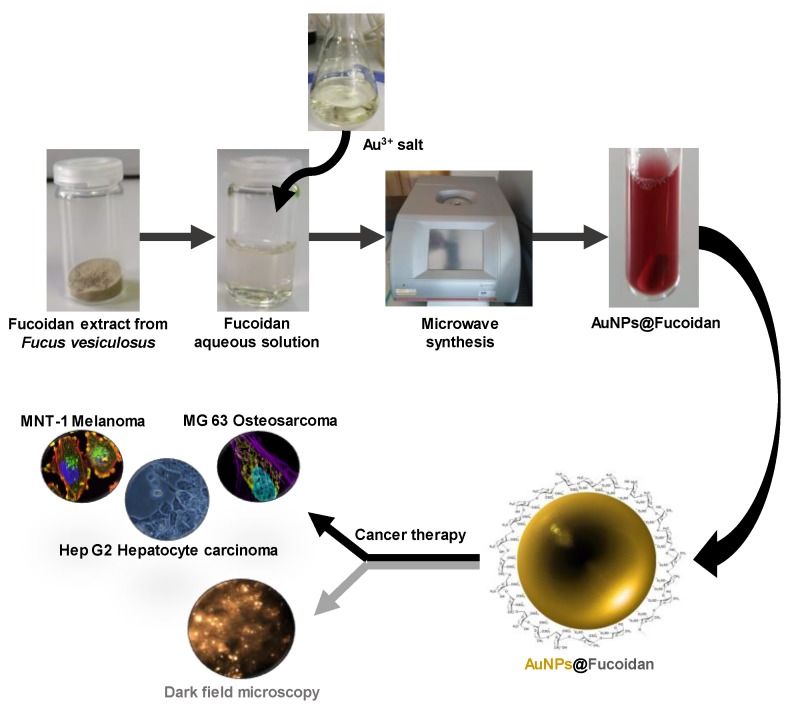
Schematic representation of the microwave irradiated synthesis of fucoidan-AuNPs (molecular structure of fucoidan) with antitumoral activity.

A fucoidan-rich fraction from *F. vesiculosus* was used as the reducing and capping agent in the green synthesis of antitumoral fucoidan-AuNPs for application in cancer therapy (Figure 1). MW technology was used for both the extraction of fucoidan from *F. vesiculosus* and the synthesis of the AuNPs, pursuing the establishment of a timesaving methodology to produce fucoidan-Au nanosystems with controllable size, morphology, and high antitumoral effect, as will be discussed in the following paragraphs.

**Figure 2:**
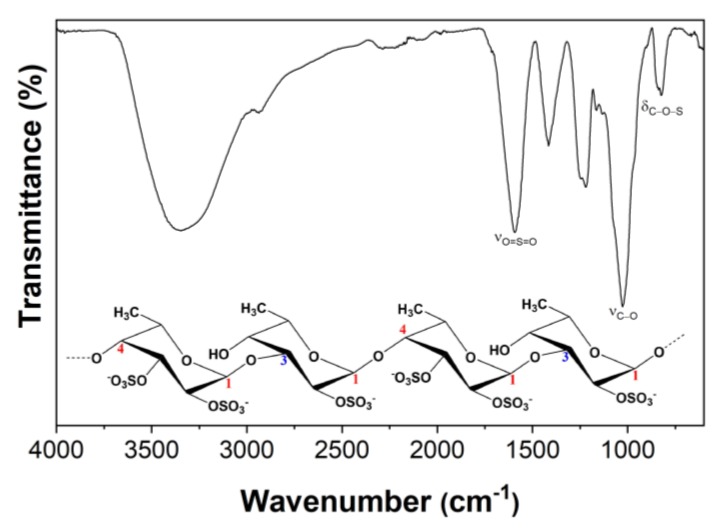
Fourier transform infrared (FTIR) spectrum of the fucoidan rich fraction extracted from *F. vesiculosus* (vibrational modes: ν = stretching, δ = bending).

The extraction yield obtained for the fucoidan-rich fraction was 5.2 ± 0.8% that is in good agreement with the published data for this algae specie under similar MAE conditions [31]. However, the degree of sulfation (5.42%) and total sugars content (13.7 ± 0.6%) are lower than those previously reported [31]. These differences are certainly associated with the natural variability of seaweed biomass [32]. The presence of fucoidan was further confirmed by FTIR analysis. The FTIR spectrum of the fucoidan-rich fraction (Figure 2) displays the typical absorption bands of fucoidans [31], namely a band at around 1260 cm^−1^ (asymmetric stretching of the O=S=O groups of sulfate esters, with the contribution of C–OH, C–C and C–O vibrations). Moreover, a band at 840 cm^−1^ (C–O–S bending associated with the axial substitution at C-4 position) and a shoulder at around 820 cm^−1^ (C–O–S bending associated with substitution at C-2 and C-3 equatorial positions of fucopyranosyl moieties) are also present [33]. The higher intensity of the band at 820 cm^−1^ suggests that the extracted fucoidan is mainly characterized by repeated units of disaccharides primarily composed of fucose residues with -OSO_3_^-^ at C-2 and C-3 positions and with a single -OSO_3_^-^ at C-2 position (Figure 2).

### 3.1. Structural and Morphological Characterization of the Fucoidan-AuNPs

**Figure 3:**
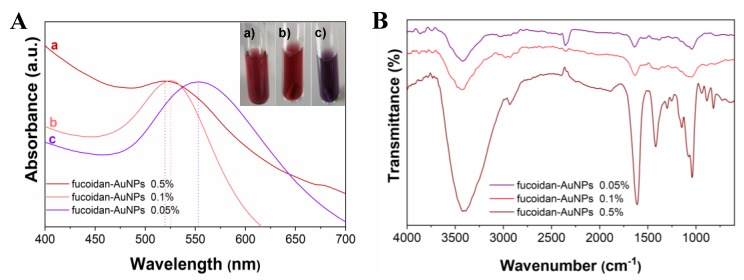
(**A**) UV-Vis and (**B**) FTIR spectra of AuNPs colloids obtained with different concentrations of fucoidan: a) 0.5%, b) 0.1%, and c) 0.05% w/v of fucoidan-rich fraction.

The MW-assisted synthesis of AuNPs, using the prepared fucoidan-rich fraction, as the reducing and capping agent, was achieved in only 1 min. Three different concentrations of fucoidan were tested, namely 0.05%, 0.1%, and 0.5% (w/v), aiming to produce AuNPs with an appropriated size and excellent colloidal stability. The color change of the Au solutions from yellowish to ruby red, for fucoidan concentrations of 0.1% and 0.5%, and to purplish for the fucoidan solution with 0.05%, is an early confirmation of the formation of the AuNPs (Figure 3A). The formation of a purple color in the case of the lowest fucoidan concentration (0.05% w/v) could be associated with the formation of larger and close particles due to the lower amount of fucoidan present at the surface of the AuNPs [21]. Additionally, the UV-vis spectra of these fucoidan-AuNPs colloids showed the typical surface plasmon resonance at around 520 nm for fucoidan concentrations of 0.1% and 0.5% (w/v). The position of these bands is indicative of the formation of small spherical NPs. The displacement of this band for higher wavelength values (around 550 nm) for the lowest fucoidan concentration (0.05%) is also in line with the formation of larger particles. Similar results were reported by Jang et al. [21] following a conventional solvothermal method but using considerably higher fucoidan concentrations (from 0.5% to 2.5% w/v).

FTIR analysis of the obtained fucoidan-AuNPs (Figure 3B) clearly confirmed the capping role of fucoidan, because of the occurrence of the typical absorption bands of fucoidan [31], as well as a correlation between the content of fucoidan in the surface of the NPs and its amount used in the synthesis.

**Figure 4:**
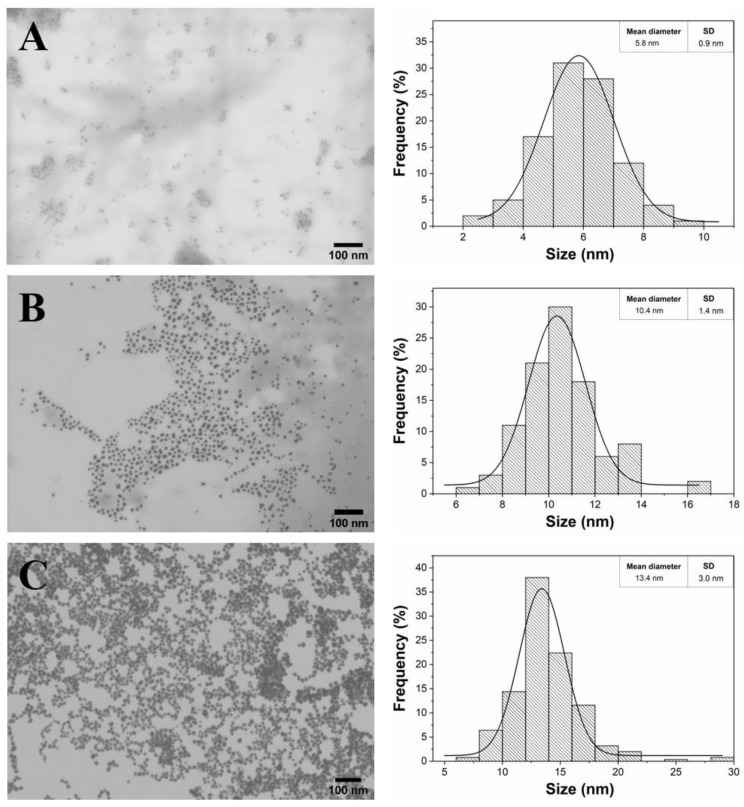
Scanning transmission electron microscopy (STEM) micrographs with the respective histograms of the size distribution of AuNPs colloids obtained with different concentrations of fucoidan: (**A**) 0.5%, (**B**) 0.1%, and (**C**) 0.05% w/v of the fucoidan-rich fraction.

The STEM micrographs provided clear information about the shape and size of the fucoidan-AuNPs (Figure 4). All the obtained fucoidan-AuNPs were monodispersed and spherical, with average sizes of 5.8 ± 0.9 nm, 10.4 ± 1.4 nm, and 13.4 ± 3.0 nm for initial concentrations of fucoidan of 0.5%, 0.1%, and 0.05% (w/v), respectively. STEM images corroborate the increase of the NPs diameter with the decrease of the fucoidan concentration. It is also perceptible that for the fucoidan concentration of 0.05%, AuNPs are closer to each other, but still individualized, because of lower colloidal stability associated with an inferior content of fucoidan on the surface of the NPs. These results are actually remarkable because in conventional solvothermal synthesis, for concentrations equal and lower than 0.5% (w/v) of fucoidan, the AuNPs formed are unstable and aggregate during synthesis leading to large anisotropic particles [21]. These results are a good indication that the MW technology allows the rapid formation of stable spherical AuNPs with controllable size and using lower concentrations of fucoidan when compared with conventional methodologies. Fucoidan-AuNPs sample with an average diameter of 10.4 ± 1.4 nm was selected for the colloidal stability studies and biological evaluations because of the monodispersity and no visual aggregation of the colloidal suspension.

### 3.2. Colloidal Stability of the Fucoidan-AuNPs

**Figure 5:**
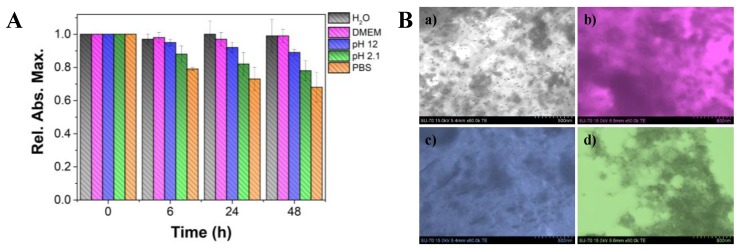
(**A**) Colloidal stability assay of fucoidan-AuNPs 0.1% (w/v) up to 48 h in distinct mediums: acid solution (pH 2.1), basic solution (pH 12), PBS, DMEM (culture medium), and ultra-purified water. (**B**) STEM images of fucoidan-AuNPs after 48 h immersed in the distinct mediums: (**a**) ultra-purified water, (b) DMEM, (c) NaOH (pH 12), and (d) HCl (pH 2.1). The color of micrographs (**b**), (**c**), and (**d**) were changed for visual guidance in order to match the color of the respective medium displayed in (A).

The colloidal stability of the fucoidan-AuNPs (10.4 ± 1.4 nm average diameter) was investigated under different conditions, namely by using acid (pH 2.1) and basic (pH 12) solutions, PBS (pH 7.4), DMEM (culture medium), and ultra-purified water, at room temperature. The stability profile over 48 h was inspected by UV-Vis and STEM analysis. The observation of the fucoidan-AuNPs colloid over time (Figure S1), and for the different conditions allowed to conclude that, in general, these NPs are considerably stable because no color changes were perceived. Based on the relative absorbance maximum obtained in UV-Vis analysis (Figure 5A), it is evident that these fucoidan-AuNPs are highly stable (more than 90% of maximum absorption) in ultra-pure water, DMEM, and alkaline solution (pH 12), with no significant variations over time. Similar results were previously reported for fucoidan-AuNPs with 15–80 nm [21] and fucoidan-Au nanorods [34]. However, in PBS (pH 7.4) and acidic (pH 2.1) solutions, the maximum absorptions slightly decrease over time, reaching about 70% and 80%, respectively. However, the analysis of the colloids by UV-Vis only gives a rough indication of the stability of the NPs because the decrease in the maximum absorption could be associated with different causes. Possible reasons are the change of the refractive index of the surrounding medium and/or the distance between the AuNPs that causes changes in the λ_máx_ of absorption (525 nm), as well as in the correspondent absorbance values [35].

To have a deep insight into the effect of the different studied conditions in the stability of the obtained colloids, in particular on their size and morphology, STEM analysis of the AuNPs after 48 h of incubation in the solutions mentioned above was also carried out. STEM micrographs (Figure 5B) confirmed that their morphology and size was not affected, but in acidic conditions, the NPs are much closer. These results indicate that under acidic conditions the fucoidan capping layer is weakened, certainly due to the protonation of sulfate groups and partial detachment from the surface of the AuNPs [36], leading to slightly less stable colloids.

### 3.3. Cytotoxicity Assays of Fucoidan-AuNPs and Cellular Uptake by Flow Cytometry

**Figure 6:**
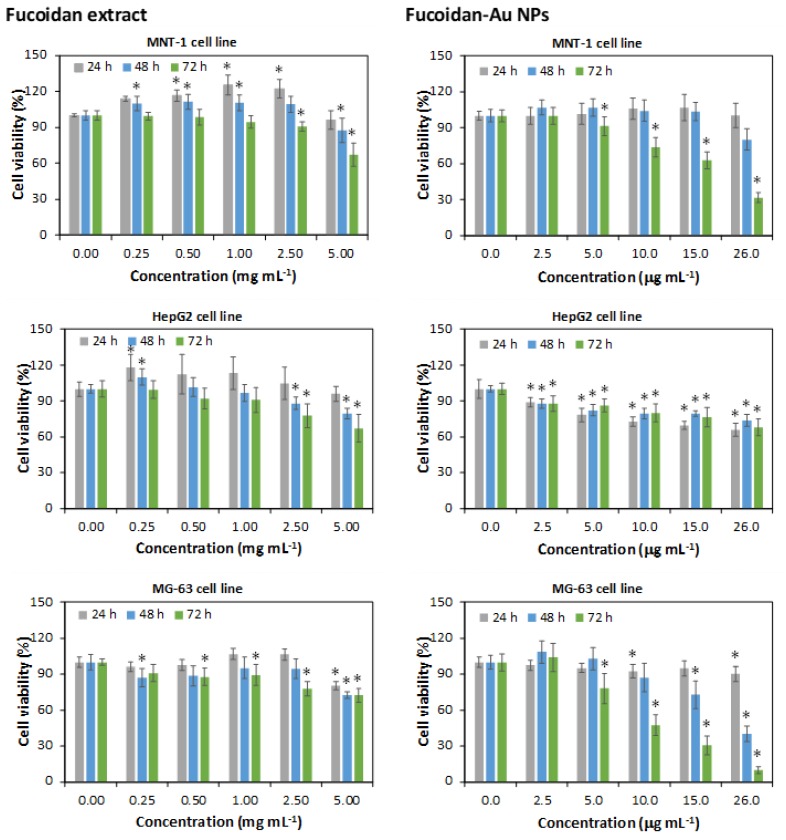
Viability measured by 3-(4,5-dimethylthiazol-2-yl)-2,5-diphenyltetrazolium bromide (MTT) assay after 24, 48, and 72 h exposure to fucoidan extract (left) and fucoidan-AuNPs (right). Values are the mean of nine replicates, and the error bars represent the standard deviation; the asterisk (*) denotes statistically significant differences to the control (p < 0.05).

In this study, the in vitro cytotoxicity of the fucoidan-enriched extract and fucoidan-AuNPs (10.4 ± 1.4 nm) was investigated against MNT-1 (pigmented human melanoma cells), HepG2 (human hepatocyte carcinoma), and MG-63 (human osteosarcoma) cell lines for 24, 48, and 72 h at concentrations ranging from 0 to 5 mg mL^−1^ (Figure 6). The fucoidan extract obtained in this study is not cytotoxic against the three cell lines tested, in the concentration range of 0.25–2.5 mg mL^−1^, with cell viabilities higher than 90% in most cases. However, for a concentration of 5 mg mL^−1^, a significant reduction of cell viability (up to 60%) was observed, particularly for 48 and 72 h of exposure. The antitumoral activity of fucoidan (and fucoidan-enriched extracts) is well documented, as well as its dependence on the source of fucoidan [15]. For instance, Manivasagan et al. [19] reported that fucoidan from *F. vesiculosus* inhibits the proliferation of human breast cancer cells with an inhibitory concentration of 35 μg mL^−1^ and Jang et al. [21] described cell viabilities of around 80% for cancer cells (HSC3) treated with 100 mg mL^−1^ of a commercial fucoidan.

The cytotoxic effect of the fucoidan-AuNPs was investigated for concentrations in the range of 2.5–26 µg mL^−1^. In general, the NPs showed a dose-depend decrease in cell viability but with noticeable differences for distinct cell lines and exposure times. For the HepG2 cell line, it was observed a reduction of cell viability with the concentration of fucoidan-AuNPs reaching about 60% cell viability for 26 µg mL^−1^ of NPs. In this case, no time dependency effect was perceived for the three exposure times investigated. For the other cell lines, it was also observed a decrease in cell viability with the concentration of NPs, but with most pronounced reductions for 48 and 72 h of exposure. For example, for a concentration of 26 µg mL^−1^ of NPs and 72 h of exposure, cell viabilities of 32 and 10% were observed for MNT-1 and MG-63 cell lines, respectively. These results demonstrate the more significant antitumoral effect of fucoidan when combined with the AuNPs because considerably higher cell viability reduction was obtained for much lower concentrations of fucoidan-AuNPs when compared with the fucoidan-enriched extract (around 60% cell viability for 5 mg mL^−1^ of fucoidan). This behavior could be associated with the small size and high surface area of the AuNPs that result in extraordinary surface concentrations of fucoidan and higher interaction with the cells. Jang et al. [21] also reported a higher cell viability reduction for fucoidan-AuNPs comparatively with fucoidan when using the same concentration of 100 mg mL^−1^.

**Figure 7:**
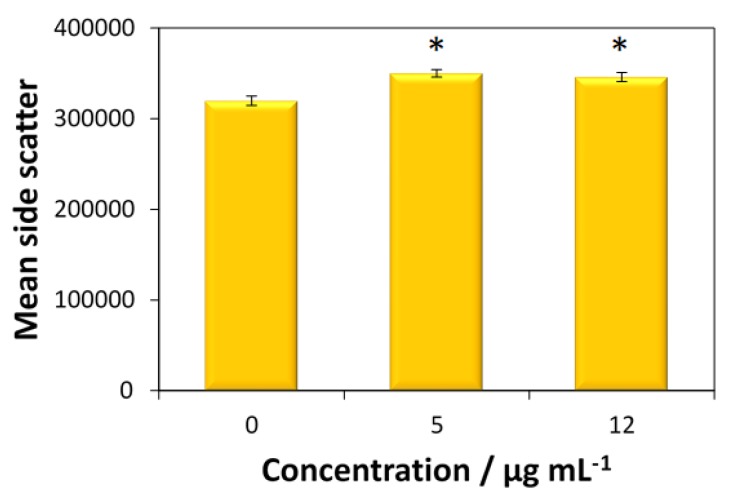
Uptake of fucoidan-AuNPs by MG-63 cells. The uptake was assessed by the side scattered light through flow cytometry after 24 h exposure to 5 and 12 μg mL^−1^ of fucoidan-AuNPs. Values are the mean of three replicates, and the error bars represent the standard deviation; the asterisk (*) denotes statistically significant differences to the control (p < 0.05).

The cellular uptake of fucoidan-AuNPs was only tested for the MG-63 cells, given the higher antitumoral activity of these nanosystems toward this cell line. According to Figure 7, both concentrations (5 and 12 μg mL^−1^) of fucoidan-AuNPs induce an increase in side scatter (SS) intensity without change of forward scatter (FS) intensity of MG-63 cells, which means that particles are internalized by the cells.

### 3.4. Dark Field Imaging of MG-63 Cells Incubated with Fucoidan-AuNPs

**Figure 8:**
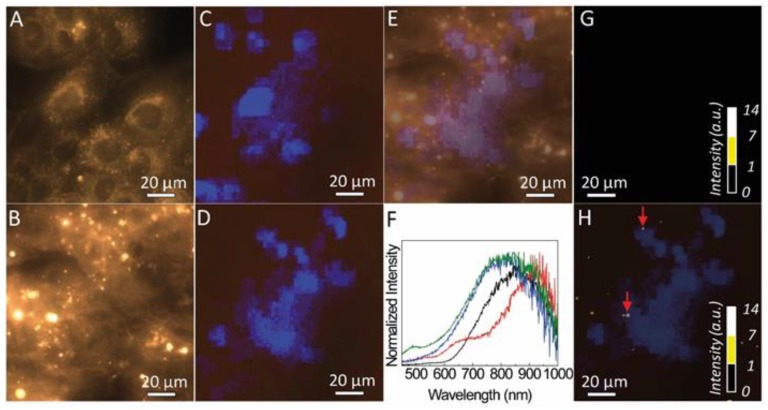
Optical images in dark field transmission mode under white light, of MG-63 cells, incubated (**A**) without and (**B**) with fucoidan-AuNPs. Optical images, in brightfield field reflectance mode under UV irradiation, of MG-63 cells incubated (**C**) without and (**D**) with fucoidan-AuNPs. (**E**) Show the overlay of (B) and (D). Spectra measured in several single pixels of the bright spots shown in the hyperspectral image shown in the (**F**). (**G**) and (**H**) show the hyperspectral images measured for the same sample and illumination conditions of (A) and (B), respectively. The color scale is based on the intensity of the spectra of each pixel at 750 nm. In (H), the hyperspectral image is superimposed with (D).

Dark field microscopy of the MG-63 cells and those incubated with fucoidan-AuNPs are shown in Figure 8A,B, respectively. The darker regions in the dark field images are assigned to the cell’s nucleus with a diameter of around 20 µm. The cells’ nucleus identification is unequivocally confirmed, taking advantage of the fact that the cells were marked with a fluorescent stain (DAPI) that binds specifically to the regions of the nucleus. Thus, under UV irradiation, blue areas are discerned (Figure 8C,D), assigned to the emission spectra around 460 nm of the DAPI (Figure S2). The overlap between the dark field images and those acquired under UV the dark areas overlap that revealing blue emission (Figure 8E). This shows that dark field imaging under white light can be used to identify the nucleus of the cells, without the need to use a fluorescent stain.

Also, the dark field images of the MG-63 cells incubated with fucoidan-AuNPs also shows bright spots with diameter values between 1 and 10 µm. The light scattering from those regions was analyzed by hyperspectral microscopy. Figure 8G,H compare the hyperspectral images of the MG-63 cells and of those incubated with fucoidan-AuNPs, revealing that the bright spots in the images of the MG-63 cells incubated with fucoidan-AuNPs are characterized by a broad spectrum. In fact, it displays a low-relative intensity band in the same region as that found for the absorption of the Au-particles (Figure 3) and a more intense one in the red spectral region, that results from the light scattered by the fucoidan-AuNPs indicating the presence of fucoidan-AuNPs aggregates (Figure 8F, Figures S3 and S4). The larger dimension of those aggregates, when compared to STEM data (Figure 4), is due to the spatial resolution of the optical image, and we also note that the contribution of the guidance of the scattered photons from the particles for the larger bright spots cannot be excluded [37]. We note that some of those bright spots (marked with arrows in Figure 8H) are localized in the same coordinates of the plane in which was possible to detect the cells, suggesting the incorporation of the fucoidan-AuNPs in the MG-63 cells, that it is in line with the flow cytometry results.

## 4. Conclusions

The one-minute microwave-assisted synthesis of fucoidan-coated gold nanoparticles (AuNPs) with controllable, high stability, and antitumoral activity is the first and foremost contribution of the present study, where fucoidan-AuNPs were synthesized by using a fucoidan-enriched fraction extracted from *F. vesiculosus*, as simultaneous reducing and capping agent. The resulting monodispersed and spherical fucoidan-AuNPs present very small diameters, namely 5.8 ± 0.9 nm, 10.4 ± 1.4 nm, and 13.4 ± 3.0 nm that depend on the initial concentrations of fucoidan: 0.5%, 0.1%, and 0.05% (w/v), respectively, together with excellent colloidal stability in acidic and basic solutions, ultra-purified water and culture media. The second innovative input of this study lies in the antitumoral activity of the fucoidan-AuNPs against three human tumor cell lines, namely MNT-1 (pigmented human melanoma), HepG2 (human hepatocyte carcinoma), and MG-63 (human osteosarcoma) cells. Moreover, the use of flow cytometry in combination with dark field imaging confirmed the cellular uptake of fucoidan-AuNPs by the MG-63 cell line, which demonstrates the antitumoral activity of these nanomaterials, and thus their potential for cancer therapy.

## Supplementary Materials

The following are available online at https://www.mdpi.com/1996-1944/13/5/1076/s1. Figure S1: Digital photographs of fucoidan-AuNPs colloid over time in the different media. Figure S2: Hyperspectral images of MG-63 cells incubated (**A**) without and (**B**) with fucoidan-AuNPs, measured for the same sample and illumination conditions of Figure 8C,D of the manuscript. The color scale is based on the intensity of the spectra of each pixel at 460 nm. (**C**) Spectra measured in collection areas of 20 × 20 pixels of (A) (red line) and (B) (blue line). Figure S3: Spectra measured in several single pixels of the hyperspectral image measured for the same sample and illumination conditions of the Figure 8A of the manuscript, before (**A**) and after (**C**) correction. (**B**) Spectrum measured in an area of 20 × 20 pixels, of the Figure 8A of the manuscript used to perform the correction. The inset in (A) shows the normalized spectra for better comparison. Figure S4: Spectra measured in several single pixels of the hyperspectral image shown in Figure 8B of the manuscript, (**A**) before and (**C**) after correction using the spectrum shown in Figure S3B. The normalized spectra before correction are shown in (**B**) for better comparison.
