# Supplementary material for: One-Minute Synthesis of Size-Controlled Fucoidan-Gold Nanosystems: Antitumoral Activity and Dark Field Imaging
Source: Materials (Basel). 2020 Feb 28;13(5):1076. doi: 10.3390/ma13051076 (PMC7084562; doi:10.3390/ma13051076)
Supplement: Supplementary file 1 [file materials-13-01076-s001.pdf]

materials

MDPI

Supplementary Material

One-Minute Synthesis of Size-Controlled Fucoidan-Gold Nanosystems: Antitumoral Activity and Dark Field Imaging

Ricardo J. B. Pinto [1,*], Daniela Bispo [1], Carla Vilela [1], Alexandre M. P. Botas [2], Rute A. S. Ferreira [2], Ana C. Menezes [3], Fábio Campos [3], Helena Oliveira [3], Maria H. Abreu [4], Sónia A. O. Santos [1] and Carmen S. R. Freire [1,*]

[1] Department of Chemistry, CICECO – Aveiro Institute of Materials, University of Aveiro, 3810-193 Aveiro, Portugal; d.bispo@ua.pt (D.B.); cvilela@ua.pt (C.V.); santos.sonia@ua.pt (S.A.O.S.)
[2] Phantom-G, Department of Physics, CICECO – Aveiro Institute of Materials, University of Aveiro, 3810-193 Aveiro, Portugal; a.botas@ua.pt (A.M.P.B.); rferreira@ua.pt (R.A.S.F.)
[3] Department of Biology & CESAM, University of Aveiro, 3810-193 Aveiro, Portugal; catarinamenezes@msn.com (A.C.M.); f.m.c@ua.pt (F.C.); holiveira@ua.pt (H.O.)
[4] ALGAplus—Prod. e Comerc. De Algas e Seus Derivados, Lda., Ílhavo, 3830-196, Portugal; helena.abreu@algaplus.pt
* Correspondence: r.pinto@ua.pt (R.J.B.P.); cfreire@ua.pt (C.S.R.F.)

Received: 06 February 2020; Accepted: 24 February 2020; Published: date

**Time /h**	0	24	48
**H_2O**	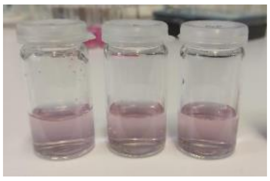	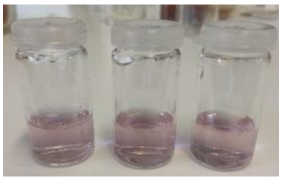	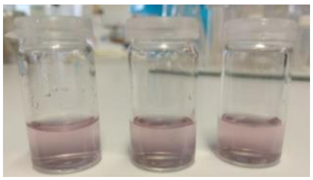
**DMEM**	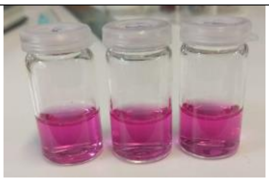	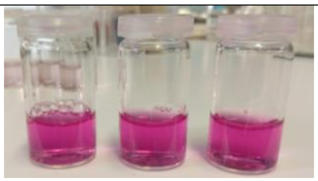	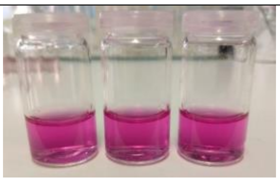
**pH 12**	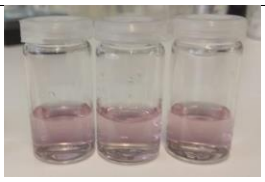	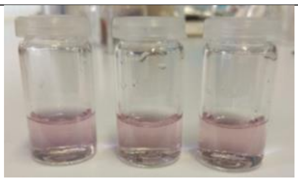	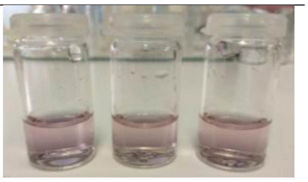
**pH 2.1**	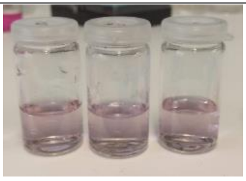	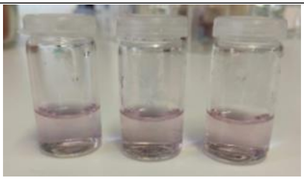	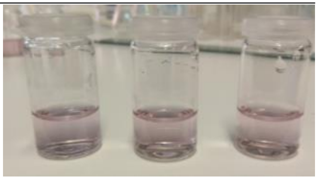
**PBS**	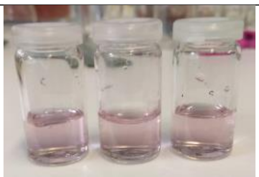	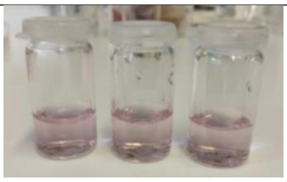	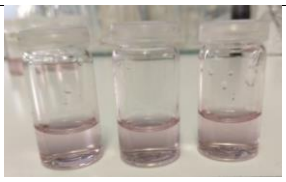

Figure S1. Digital photographs of fucoidan-Au NPs colloid over time in the different media.

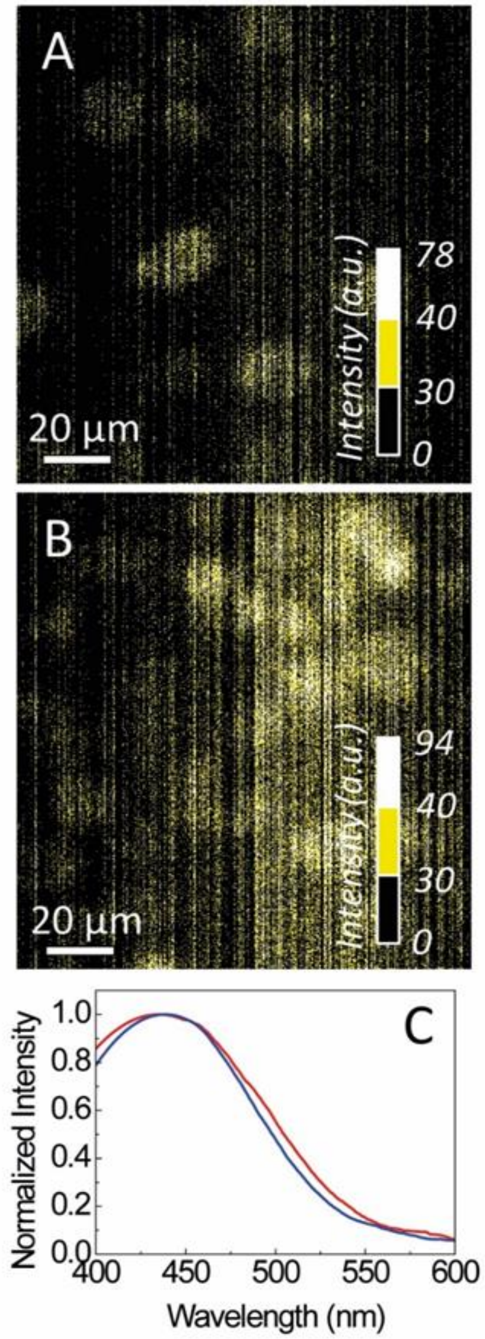

Figure S2. Hyperspectral images of MG-63 cells incubated (**A**) without and (**B**) with fucoidan-Au NPs, measured for the same sample and illumination conditions of Figure 8C and 8D of the manuscript. The color scale is based on the intensity of the spectra of each pixel at 460 nm. (**C**) Spectra measured in collection areas of 20×20 pixels of (**A**) (red line) and (**B**) (blue line).

Correction of the spectra measured by hyperspectral microscopy

Figure S3A shows selected spectra measured at distinct single pixels of the hyperspectral image shown in Figure 8G of the manuscript. The spectra are formed by a broad band between 500 to 800 nm and were corrected using the spectrum measured at a region of the same image (Figure S3B). The correction allows removing from the spectrum of each pixel the profile of the spectrum shown in Figure S2B. The spectra that result from the correction are shown in Figure S3C. The profile of the corrected spectra presents an intensity of approximately zero, which indicates that the spectral profile of each pixel is identical to the spectral profile of the spectra used to perform the correction. This shows that there are no remarkable differences between the scattering features of the structures

shown in Figure 8A of the manuscript, and allows to state that besides the MG-63 cells no other structure contributes significantly to the scattering, since the elimination from the spectra of the scattering band of the MG-63 cells, results in a scattering intensity close to zero.

Figure S4A and Figure S4B show the spectra measured at distinct single pixels of the hyperspectral image shown in Figure 8H of the manuscript. The spectra present differences not only in the scattering intensity but also in the spectral profile. The spectra were corrected using the spectrum shown in Figure S3B, which allows removing the contribution of the scattering of the MG-63 cells from the spectrum measured at each pixel of the image of the MG-63 cells incubated with fucoidan-Au NPs. After correction, some spectra present an intensity of approximately zero (Figure S4C), indicating that the scattering of light by the structure that corresponds to this pixel comes from the scattering by the MG-63 cells. Other corrected spectra still present a broad scattering band after removing the contribution of the scattering from the MG-63. This indicates that these spectra were measured in pixels in which besides the MG-63, there is also another structure, such as the fucoidan-Au NPs that present a scattering feature that differs from the MG-63 cells. The fact that the bright spots in Figure 8H of the manuscript present more scattering of light and have a distinct scattering profile when compared with the scattering profile of the MG-63 cells is in good agreement with the idea that the bright spots in Figure 8B of the manuscript corresponds to fucoidan-Au NPs. The existence of scatting signal for the fucoidan-Au NPs with distinct profiles can be explained by the dependence on the scattering profile on the size of the NPs and on the size of the agglomerates of NPs.

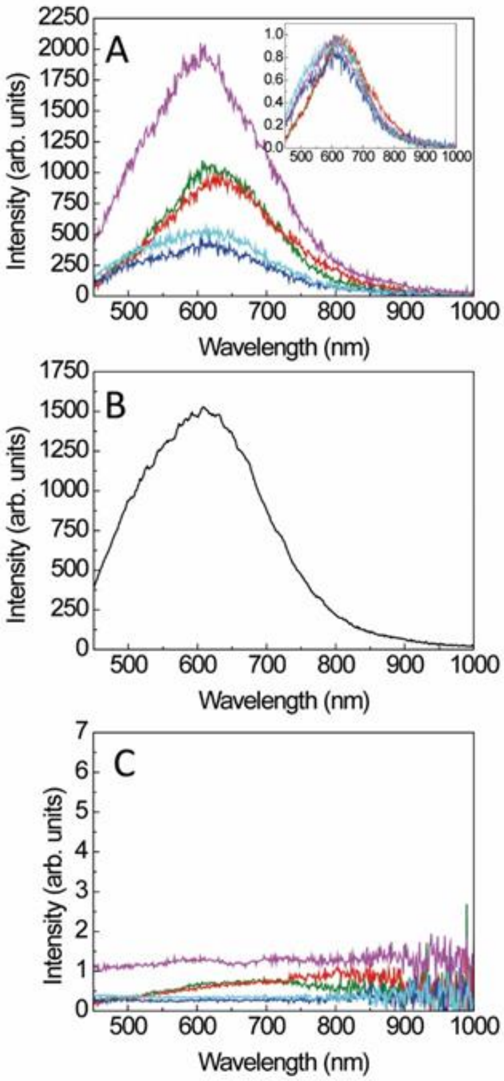

Figure S3. Spectra measured in several single pixels of the hyperspectral image measured for the same sample and illumination conditions of the Figure 8A of the manuscript, (**A**) before and (**C**) after correction. (**B**) Spectrum measured in an area of 20×20 pixels, of the Figure 8A of the manuscript used to perform the correction. The inset in (**A**) shows the normalized spectra for better comparison.

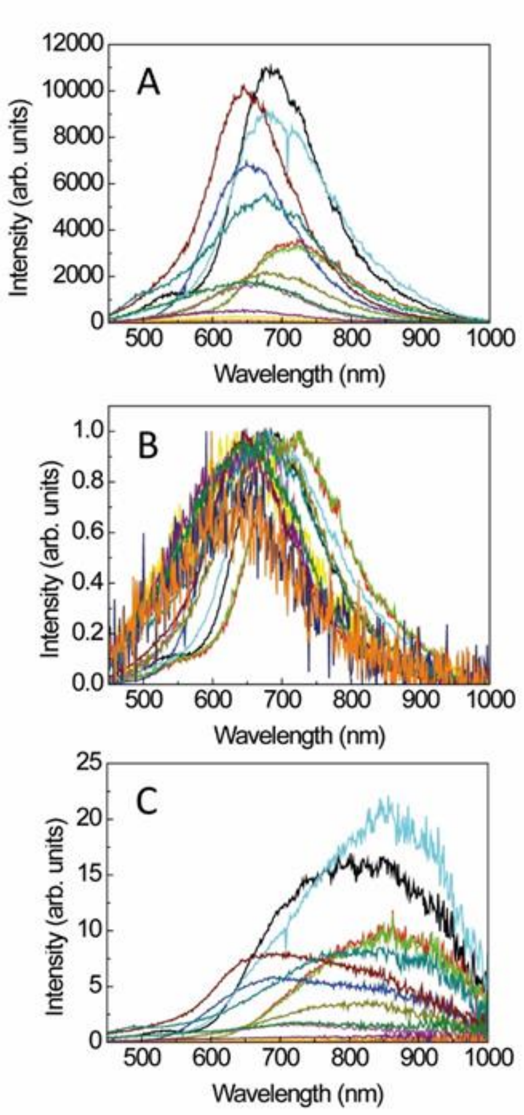

Figure S4. Spectra measured in several single pixels of the hyperspectral image shown in Figure 8B of the manuscript, (**A**) before and (**C**) after correction using the spectrum shown in Figure S3B. The normalized spectra before correction are shown in (**B**) for better comparison.

© 2020 by the authors. Submitted for possible open access publication under the terms and conditions of the Creative Commons Attribution (CC BY) license (http://creativecommons.org/licenses/by/4.0/).
